# Seroprevalence of *Toxoplasma gondii* infection in slaughtered chickens, ducks, and geese in Shenyang, northeastern China

**DOI:** 10.1186/1756-3305-5-237

**Published:** 2012-10-18

**Authors:** Na Yang, Ming-Yang Mu, Hong-Kui Li, Miao Long, Jian-Bin He

**Affiliations:** 1 College of Animal Science and Veterinary Medicine, Shenyang Agricultural University, Shenyang, Liaoning Province 110866, China; 2 Liaoning Center for Animal Disease Control and Prevention, Shenyang, Liaoning Province, 110164, China

**Keywords:** *Toxoplasma gondii*, Seroprevalence, Chicken, Duck, Goose, MAT

## Abstract

**Background:**

In recent years, investigations of *Toxoplasma gondii* infection in poultry (chickens, ducks, and geese) have been reported worldwide, including China. However, little is known about the prevalence of *T. gondii* infection in poultry in northeastern China. Therefore, the present study was performed to determine the prevalence of *T. gondii* infection in slaughtered chickens, ducks, and geese in Shenyang, northeastern China.

**Methods:**

In the present study, the seroprevalence of *T. gondii* in 502 adult chickens, 268 adult ducks, and 128 adult geese was surveyed using the modified agglutination test (MAT).

**Results:**

The seroprevalences of *T. gondii* were 5.8%, 7.8%, and 4.7% in chickens, ducks, and geese, respectively. Prevalence was higher in free-range groups (11.2%, 12.3%, and 8.9%) than caged groups (4.7%, 7.5%, and 6.0%), and there was a statistically significant difference only between free-range chickens and caged chickens, but no significant difference was found between free-range ducks, geese and caged ducks, geese.

**Conclusions:**

The present study shows the prescence of *T. gondii* infection in slaughtered chickens, ducks, and geese in Shenyang, northeastern China, which suggests that consumption of poultry meat in Shenyang may pose a potential threat to human health and should be given attention.

## Background

Toxoplasmosis is a widely prevalent zoonotic disease, caused by *Toxoplasma gondii*[1-5]. Domestic cats and other felids are the only definitive hosts for the parasite, and almost all warm-blooded animals and birds, including humans, are intermediate hosts. Humans can be infected by ingestion of raw, or undercooked, meat from the infected animals, and by consuming food or water contaminated with oocysts excreted by cats [1]. Meat from *T. gondii*-infected poultry (including chickens, ducks, geese, and pigeons) is consumed widely in many countries, including China, and is known to be the primary source of infection for humans [1,6]. Moreover, the prevalence of *T. gondii* in chickens and ducks is a good indicator of soil contamination with *T. gondii* oocysts [7].

Worldwide seroprevalences of *T. gondii* in chickens, ducks, and geese are summarized by Dubey [1]. In recent years, there have several reports of *T. gondii* infection in chickens, ducks, and geese in China [8-11]. However, there is no information regarding *T. gondii* infection in ducks and geese, and only limited information on seroprevalence of *T. gondii* in chickens in Liaoning, China, therefore, an investigation of the seroprevalence of *T. gondii* infections in chickens, ducks, and geese was undertaken.

## Methods

### The study area

The study was conducted in Shenyang City, the capital of Liaoning Province, northeastern China. Shenyang is located in the southern part in northeastern China, covering an area of 1, 2948 km^2^ and a population of approximately 8.19 million. Its geographical position is at east longitude 122°25^′^ - 123°48^′^ and at north latitude 41°11^′^ - 43°2^′^. The area has a temperate monsoon climate, with abundant sunshine, a long winter and summer, with a brief spring and autumn. The average annual temperature is 8.3°C, with a mean annual rainfall of 600–800 mm.

Three different poultry abattoirs located in Dadong, Heping, and Shenbei in Shenyang, were selected for sample collections. All of the above abattoirs are the main suppliers of poultry meat to Shenyang and the neighboring regions.

### Blood samples

A total of 502 blood samples from adult chickens, 268 blood samples from adult ducks, and 128 blood samples from adult geese were collected from the above three poultry abattoirs in Shenyang between February and July 2012. Free range birds and caged birds were separated to slaughter in the same abattoirs and sold to market. The blood samples were sent to the laboratory for serological examination and centrifuged at 3,000 rpm for 10 min, and the sera were stored at −20°C until tested for antibodies to *T. gondii*.

### Serological assay

Sera were tested for *T. gondii* antibodies using 2-fold serial dilutions from 1:25 to 1:3,200 with the modified agglutination test (MAT), as described previously [12]. Briefly, the harvested parasites were kept in 6% formaldehyde solution at 4°C overnight, and suspended in the alkaline buffer at 20,000 parasites/mL. Two-fold dilutions of sera were performed using the serum diluting buffer, and agglutination was performed in U-bottom 96-well microtiter plates using a mixture of 50 μL antigen and 50 μL diluted sera. The plates were incubated at 37°C overnight. The test was considered positive when a layer of agglutinated parasites was formed in wells at dilutions of 1:25 or higher; positive and negative controls were included in each test.

### Statistical analysis

Statistical analysis of *T. gondii* prevalence between free-range (FR) and caged groups was performed using a Chi square test with SPSS (SPSS Inc., Chicago, Illinois). A *P*-value < 0.05 was considered statistically significant.

### Ethics statement

All animals were handled in strict accordance with good animal practice according to the Animal Ethics Procedures and Guidelines of the People's Republic of China, and the study was approved by the Animal Ethics Committee of Shenyang Agricultural University (Permit No. SYXK<Liao>2011-0001).

## Results and discussion

Overall, the seroprevalences of *T. gondii* were 5.8%, 7.8%, and 4.7% in chickens, ducks, and geese, respectively (Table 1).

**Table 1:** **Seroprevalence of *Toxoplasma gondii *infection in chickens, ducks, and geese in Shenyang, northeastern China by the modified agglutination test (MAT)**

**Animal**		**No. tested**	**No. with anti-*T. gondii* antibodies**					**Total**	**Prevalence**
**species**			**1:25**	**1:50**	**1:100**	**1:200**	**1:400**	**positive**	**(%)**
Chicken	FR	206	16	4	0	0	3	23	11.2
	Caged	296	13	1	0	0	0	14	4.7
	Total	502	29	5	0	0	3	37	5.8
Duck	FR	122	12	1	1	1	0	15	12.3
	Caged	146	9	2	0	0	0	11	7.5
	Total	268	21	3	1	1	0	26	7.8
Goose	FR	45	2	2	0	0	0	4	8.9
	Caged	83	4	0	0	1	0	5	6.0
	Total	128	6	2	0	1	0	9	4.7

*FR*: Free-range.

In the present study, 37 (5.8%) of 502 chickens were seropositive to *T. gondii*, with titers of 1:25 in 29, 1:50 in 5, and 1:400 in 3 (Table 1). The seroprevalence of *T. gondii* from 3 different abattoirs ranged from 6.2% to 8.9% (Table 2). High prevalence was found in FR chickens (11.2%), compared with caged chickens (4.7%) (χ^2^ = 7.37, P<0.01), indicating that FR chickens are more likely to be infected by *T. gondii* oocysts since they feed on the ground. The present study showed that the overall seropositivity (5.8%) for *T. gondii* infection in chickens was lower than those tested in other countries [13]. In China, it was also lower than the 25.2% prevalence reported for chickens in a study conducted in Jinzhou [11], 8.4% in Guangzhou [8], 7.4% in Zhangjiakou [9], and 7.3% in Lanzhou [14].

**Table 2:** **Seroprevalence of *Toxoplasma gondii *infection in chickens, ducks, and geese in different poultry abattoirs in Shenyang, northeastern China by the modified agglutination test (MAT)**

**Animal species**	**Poultry abattoir**	**No. tested**	**No. positive**	**Prevalence (%)**
Chicken	Dadong	167	11	6.6
	Heping	145	9	6.2
	Shenbei	190	17	8.9
Duck	Dadong	101	10	9.9
	Heping	79	9	11.4
	Shenbei	88	7	8.0
Goose	Dadong	45	3	6.7
	Heping	24	2	8.3
	Shenbei	59	4	6.8

Antibodies to *T. gondii* were found in 26 of 268 (7.8%) ducks with titres of 1:25 in 21, 1:50 in 3, 1:100 in 1, and 1:200 in 1 (Table 1). The seroprevalence in the present study was lower than those reported in other countries [1], and also lower than 16.0% in Guangzhou [8], 11.4% in Lanzhou [14] in China. The seroprevalence (12.3%) in FR ducks was higher than 7.5% in caged ducks, but no significant difference was found between FR ducks and caged ducks. Seroprevalence of *T. gondii* infection from 3 poultry abattoirs ranged from 8.0% to 11.4% (Table 2).

*T. gondii* antibodies were detected in 9 (4.7%) of 128 tested geese with titers of 1:25 in 6, 1:50 in 2, and 1:200 in 1 (Table 1), and the seroprevalences in FR and caged geese were 8.9% and 6.0%, respectively, but the difference was not statistically significant (P>0.05). To our knowledge, there was only one report regarding *T. gondii* infection in geese in Guangdong, China [10], and the seroprevalence (4.7%) in Shenyang in this study was lower than 15.0% in Gungdong. Seroprevalence of *T. gondii* infection from 3 poultry abattoirs ranged from 6.7% to 8.3% (Table 2).

The seroprevalences in chickens, ducks, and geese, were lower than those in other regions in China, which may be affected by ecological and geographical factors, as well as feeding conditions. The average annual temperature in Shenyang is 8.3°C, a rather cold climate, with typically dry conditions, which may be unfavorable for the survival of *T. gondii* oocysts.

The modified agglutination test (MAT) has been evaluated extensively in experimentally and naturally infected birds, and is sensitive and specific for assay of *T. gondii* antibodies [1,10,13,14], compared to other serologic methods. However, the test needs to be validated by bioassays of viable *T. gondii* from meat consumed by humans in China.

## Conclusions

The present study shows the *T. gondii* infection in slaughtered chickens, ducks, and geese in Shenyang, northeastern China, which suggests that consumption of poultry meat in Shenyang may pose a potential threat to human health and should be given attention.

## Competing interests

The authors declare that they have no competing interests.

## Authors’ contributions

JBH and NY conceived and designed the study, and critically revised the manuscript. NY, MYM, HKL and ML performed the experiments, analysed the data and drafted the manuscript. All authors read and approved the final manuscript.
